# Lack of Evidence for a Role of Islet Autoimmunity in the Aetiology of Canine Diabetes Mellitus

**DOI:** 10.1371/journal.pone.0105473

**Published:** 2014-08-25

**Authors:** Kerstin M. Ahlgren, Tove Fall, Nils Landegren, Lars Grimelius, Henrik von Euler, Katarina Sundberg, Kerstin Lindblad-Toh, Anna Lobell, Åke Hedhammar, Göran Andersson, Helene Hansson-Hamlin, Åke Lernmark, Olle Kämpe

**Affiliations:** 1 Department of Medical Sciences, Science for Life Laboratory, Uppsala University, Uppsala, Sweden; 2 Department of Medical Sciences, Molecular Epidemiology and Science for Life Laboratory, Uppsala University, Uppsala, Sweden; 3 Department of Immunology, Genetics and Pathology, Uppsala University, Sweden; 4 Department of Clinical Sciences, Swedish University of Agricultural Sciences, Uppsala, Sweden; 5 Department of Animal Breeding and Genetics, Swedish University of Agricultural Sciences, Uppsala, Sweden; 6 Broad Institute of Harvard and MIT, Cambridge, Massachusetts, United States of America; 7 Science for Life Laboratory, Department of Medical Biochemistry and Microbiology, Uppsala University, Uppsala, Sweden; 8 Diabetes and Celiac Disease Unit, Department of Clinical Sciences, Lund University, Malmö, Sweden; St. Vincent's Institute, Australia

## Abstract

**Aims/Hypothesis:**

Diabetes mellitus is one of the most common endocrine disorders in dogs and is commonly proposed to be of autoimmune origin. Although the clinical presentation of human type 1 diabetes (T1D) and canine diabetes are similar, the aetiologies may differ. The aim of this study was to investigate if autoimmune aetiology resembling human T1D is as prevalent in dogs as previously reported.

**Methods:**

Sera from 121 diabetic dogs representing 40 different breeds were tested for islet cell antibodies (ICA) and GAD65 autoantibodies (GADA) and compared with sera from 133 healthy dogs. ICA was detected by indirect immunofluorescence using both canine and human frozen sections. GADA was detected by *in vitro* transcription and translation (ITT) of human and canine GAD65, followed by immune precipitation. Sections of pancreata from five diabetic dogs and two control dogs were examined histopathologically including immunostaining for insulin, glucagon, somatostatin and pancreas polypeptide.

**Results:**

None of the canine sera analysed tested positive for ICA on sections of frozen canine or human ICA pancreas. However, serum from one diabetic dog was weakly positive in the canine GADA assay and serum from one healthy dog was weakly positive in the human GADA assay. Histopathology showed marked degenerative changes in endocrine islets, including vacuolisation and variable loss of immune-staining for insulin. No sign of inflammation was noted.

**Conclusions/Interpretations:**

Contrary to previous observations, based on results from tests for humoral autoreactivity towards islet proteins using four different assays, and histopathological examinations, we do not find any support for an islet autoimmune aetiology in canine diabetes mellitus.

## Introduction

Diabetes mellitus occurs in dogs in Sweden with an incidence of 13 cases per 10,000 years-at-risk and a mean age of onset at 8.6 years [1]. The domestic dog shares its environment and lifestyle with its owner and has a breed structure that is highly suitable for genetic analysis [2]. A prerequisite for comparative genetic studies is, however, a common aetiology of disease.

There is no internationally accepted classification system for canine diabetes mellitus but the aetiology has been broadly divided into primary insulin resistance or primary insulin deficiency diabetes. According to this classification, canine insulin resistance is not depicting a primary cellular peripheral insulin resistance, but can occur as a consequence of diverse hormonal disturbances. Furthermore, canine insulin deficiency diabetes, universally claimed to resemble latent autoimmune diabetes in the adult, has suggested to be a result of autoimmunity or in some cases, secondary to exocrine pancreatic disease [3], [4]. Different breeds could be predisposed to different form of diabetes mellitus as shown for the Swedish and Norwegian elkhounds, which develop a reversible form of diabetes during pregnancy or pseudopregnancy, resembling human gestational diabetes mellitus [5].

Findings in pancreatic biopsy samples from diabetic dogs have been conflicting. Two fairly large studies of diabetic dogs [6], [7] (n = 33, n = 30) have shown a reduced number or total absence of islets, together with degeneration, hyalinisation or vacuolisation. Pancreatic biopsy samples from dogs with diabetes mellitus secondary to hormonal disturbances also showed degeneration and vacuolisation of the beta cells [8] No lymphocytic infiltration was seen in any of these three studies. On the other hand, Alejandro et al reported that 6/13 diabetic dogs in their study had lymphocytic infiltration associated with islets (insulitis) and that 5/18 dogs displayed extensive exocrine pancreatic damage [9]. There were no control groups in any of these studies.

The occurrence of autoantibodies is a central characteristic of human T1D, and islet cell antibodies (ICA) and GAD65 autoantibodies are valuable diagnostic markers. The presence of autoantibodies in canine diabetes mellitus remains controversial, since both presence and absence of autoantibodies have been reported [5], [9]–[12]. Insulitis is commonly reported in samples from recent onset human T1D subjects [13]. In this study we investigated sera from dogs with and without diabetes using four different assays in addition to histopathological examination of diabetic and control pancreata, with the aim to investigate if autoimmune aetiology resembling human T1D is prevalent in dogs.

## Methods

### Dogs

Serum samples were obtained from privately owned diabetic (*n* = 121) and healthy control (*n* = 133) dogs of 64 breeds in total (60% female) [14] (Table 1). In addition, sera from dogs diagnosed with adrenocortical insufficiency (*n* = 5) and lymphocytic thyroiditis (*n* = 13) were included. The diagnosis of diabetes mellitus in cases was based on the clinical picture and chronic fasting hyperglycaemia. The mean age at diagnosis of diabetes was 8.2 years (SD 1.9). Samples for analysis of autoantibodies were taken at a median of 88 days after diagnosis of DM (IQR 11-448 days).

**Table 1:** Breed and disease status of dogs analysed in the study.

Breed	Diabetic	Healthy	Lymphocytic Thyroiditis	Adrenal insufficiency
**Afghan hound**		1		
**Airedale terrier**		1		
**Australian cattle dog**	1			
**Australian terrier**	6			
**Basenji**		1		
**Basset artesian normand**		2		
**Beagle**		17		
**Bearded collie**				1
**Border collie**	9	2		
**Border terrier**	2	1		
**Borzoi**	1			
**Boxer**		6		1
**Bullmastiff**	1	1		
**Cairn terrier**	3			
**Cavalier king charles spaniel**	1			
**Cocker spaniel**	1	1		
**Collie**		2		
**Dachshund**	9			
**Danish/Swedish farm dog**	1			
**Doberman pinscher**	1			
**Drever (Swedish dachsbracke)**	2			
**English springer spaniel**	1	3		
**Finnish hound**		2		
**Finnish lapphound**	1			
**Finnish spitz**		1		
**Flat-coated retriever**		2		
**Fox terrier**		2		
**German shepherd dog**	1	5		
**Giant schnauzer**	1	9	13	
**Golden retriever**		8		
**Hollandse herder (Dutch shepherd)**	1			
**Hovawart**		1		
**Irish setter**	1	1		
**Irish wolfhound**		3		
**Jack Russell terrier**		4		
**Labrador retriever**	5	2		
**Laika**		1		
**Lapp hound**	2			
**Medium poodle**		2		
**Miniature poodle**	2			
**Mixed breed**	9	4		
**Norwegian elkhound**		7		
**Nova Scotia duck tolling retriever**		3		3
**Papillion**		1		
**Petit brabancon**	1			
**Polski owczarek nizinny**	6	2		
**Poodle**	2			
**Portuguese waterdog**	1			
**Rhodesian ridgeback**		1		
**Rottweiler**	5	2		
**Saluki**	1			
**Samoyed**	2			
**English Setter**	1	1		
**Schipperke**	2	1		
**Staffordshire bull terrier**	1			
**Standard poodle**		1		
**Swedish elkhound**	26	22		
**Swedish spitz (västgötaspets)**	1			
**Swedish/Norwegian elkhound**	1			
**Tervueren**		3		
**Tibetan terrier**	2			
**Vorsteh**		2		
**West highland white terrier**	7	2		
**Sum**	**121**	**133**	**13**	**5**

In a subset (*n* = 51) of the dogs, a glucagon stimulation test with C-peptide measurements was performed [14], which turned out negative for the vast majority of dogs indicating an insulin deficiency-type of diabetes mellitus. Pancreata were available from five dogs of different breeds diagnosed with diabetes (4 females and 1 male). Dogs were euthanized at the time of diagnosis. Specimens from two normoglycemic dogs euthanized for other reasons than diabetes served as controls. The study was approved by the Uppsala animal ethics committee (C 267/5) and all dog owners gave informed written consent.

### Pathology

Tissue specimens were fixated in 10% buffered neutral formalin at room temperature, followed by routine processing to paraffin wax. Approximately 4-µm thick sections were cut and attached to positively charged glass slides (IHC Microscopic Slides, Flex (DAKO, Glostrup, Denmark).The sections were microwave treated for 2×5 min at 700 W by using 50 mM Tris buffer saline, as retrieval solution and immunostained by using a polymer detection system, Dako Cytomation, EnVision+ System-HRP; K4010 for primary antibodies and K4006 for primary mouse antibodies. The incubation time was 30 min at room temperature. Diaminobenzidine was used as chromogen. The antibodies were diluted in Dako's antibody diluent. The following antibodies were used: Insulin (mouse monoclonal, diluted 1∶2000, K36AC10, Sigma-Aldrich), Glucagon (mouse monoclonal, 1∶10000, K79bB10, Abcam), Somatostatin (rabbit polyclonal, 1∶4000, A0566, Dako), pancreas polypeptide (rabbit polyclonal, 1∶10000, A0619, Dako). Meyer's hematoxylin from Histolab (Gothenburg, Sweden) was used for nuclear counterstain. Negative controls, excluding the primary antibody, were used.

### Immunostainings for presence of anti-islet antibodies in sera

Pancreata were dissected from dogs euthanized for reasons other than endocrine and pancreatic disorders and snap frozen in liquid nitrogen. Six µm thick cryostat sections were placed on glass slides (Superfrost plus, Thermo Scientific, Braunschweig, Germany), air-dried and blocked for unspecific binding for 30 min in room temperature using 10% normal goat sera (Dako, Glostrup, Denmark) in phosphate buffered saline (PBS) pH 7.4, containing 14% aprotinin (Trasylol, Bayer, Germany). The immunostainings were performed over night at +4°C, using 100 µl serum/glass from every diabetic and control dog individually –diluted in the range 1∶16 to 1∶64. Three positive controls were used, including sera from a human diabetes patient, sera from a patient with stiff person syndrome and a monoclonal mouse GAD antibody (BD Biosciences, San Jose, CA, USA) diluted to 1∶200. The slides were rinsed three times in PBS and incubated with 100 µl of either secondary pre-adsorbed, FITC conjugated goat anti dog IgG (Santa Cruz Biotechnology, CA, USA), FITC conjugated goat anti human IgG (H+L) or Cy3 conjugated goat anti human IgG and Cy3 conjugated goat anti mouse IgG (all three from Jackson Immuno Research, West Grove, PA, USA) in 1∶200 dilutions. After one h incubation at room temperature the slides were rinsed five times in PBS and mounted with Vectashield (Vector Laboratories, Burlingame, CA, USA) with or without nuclear staining 4′,6-diamidino-2-phenylindole (DAPI). All incubations were performed in a covered humid chamber. The presence of Ab staining was assessed in a Nikon microphot–FXA fluorescence microscope. For documentation images were also taken using a Zeiss LSM 501 confocal microscope. The human ICA assay was performed by an accredited laboratory at Malmö Academic Hospital according to standard procedures.

### *In-vitro* transcription and translation with canine and human GAD65 followed by immunoprecipitations

*In-vitro* transcription and translation (ITT) of canine GAD65 was performed using the TNT-SP6 quick coupled transcription/translation system (Promega, Madison, WI, USA) with the addition of 0.5 mCi of [^35^S]-methionine (10 µCi/µl, Perkin Elmer, Waltham, MA, USA) as previously described [5]. Approximately 20 000 cpm of the [^35^S]-radio labelled ITT products were used for immunoprecipitation with 2.5 µl serum. Immunoprecipitations of the labelled protein with canine sera were performed using protein A Sepharose (GE Healthcare Biosciences, Uppsala, Sweden) followed by measurement of the radioactivity. Two GADA-positive human sera were used as positive controls. Furthermore, a clone encoding the human protein GAD65 was used as above. A human GADA-positive serum and BSA were used as positive and negative controls. The antibody reactivity was calculated as indices relative to a positive (human serum) and negative control (4% BSA) according to the following equation: Immunoreactivity index  =  ((cpm subject x-cpm negative standard)/(cpm positive standard -cpm negative standard) * 100). Sera with an immunoreactivity index exceeding the mean value for healthy dogs (*n* = 133) plus 4 standard deviations were regarded as positive.

## Results

### Pathology

**Figure 1:**
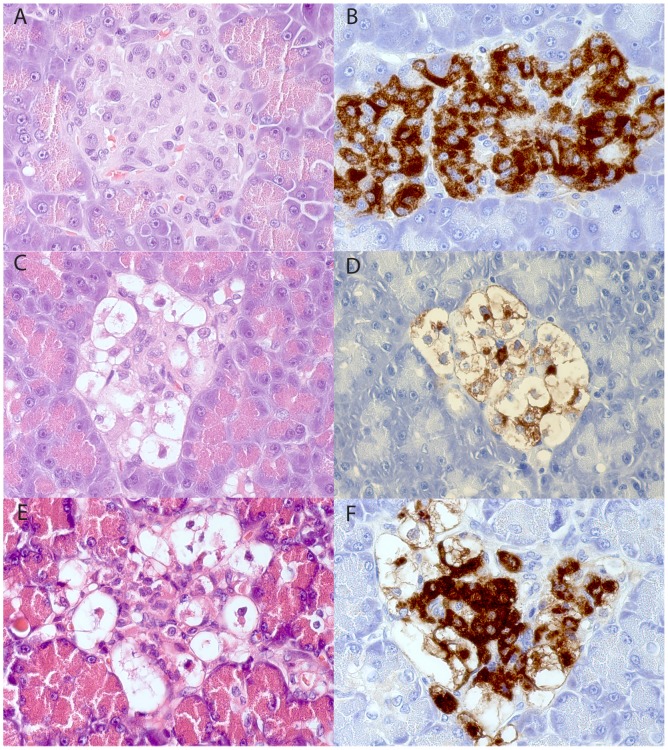
Pancreatic specimens stained with hematoxylin-eosin (A, C, E) and immunostained for insulin (B, D, F). The samples shown in A and B come from a non-diabetic male Swedish Elkhound, C and D come from a diabetic male Polish Owczarek Nizinny dog and E and F from a diabetic female English Setter. The islet in Fig D contains few insulin-immunoreactive cells except the vacuolated cells, while these normal-appearing insulin-stained cells are numerous in Fig F.

The frequency and distributions of islets varied in the diabetic animals as well as in the non-diabetics. Generally the islet sizes in both groups were smaller compared to that observed in human pancreas. No inflammatory reaction was seen in any of the animal pancreata. There were no signs of degeneration or atrophy in the exocrine parenchyma. The islets in both animal groups contained all four endocrine cell types but a large fraction of the insulin-staining (beta) cells in the diabetic group were enlarged, vacuolated with no or only traces of insulin-immunoreactivity (Fig 1A–F). Non-vacuolated normal appearing insulin-stained cells were present in all diabetic pancreata with a varying frequency from rare to frequent. Images from all seven dogs with HE, insulin and glucagon staining is available in the Supplementary Material (Fig. S1).

### Immunostainings for presence of anti-islet antibodies in sera

**Figure 2:**
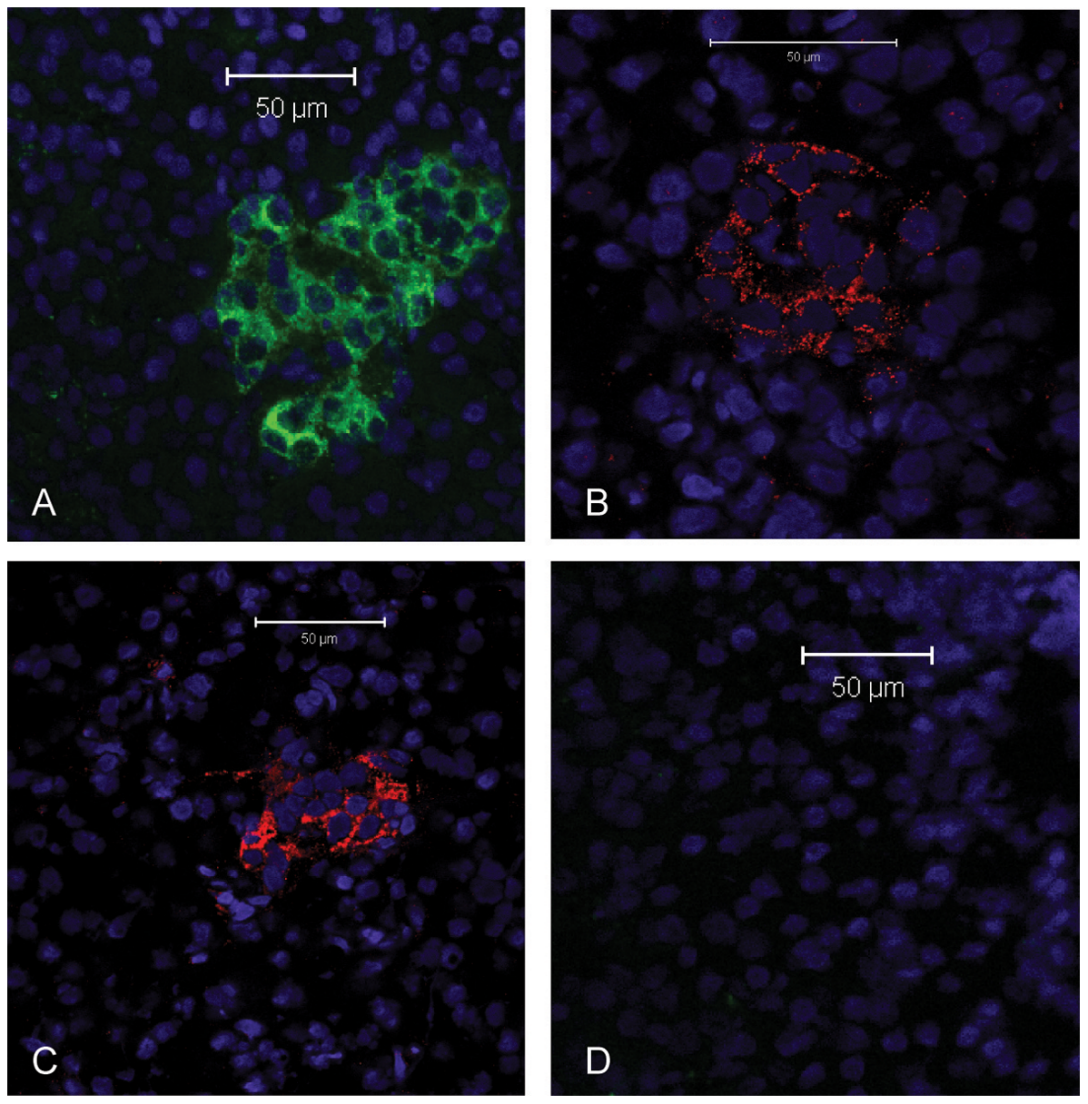
Canine pancreas stained by immunofluorescence. A) Stained with a mouse monoclonal Ab to human GAD65, B) stained using a GADA positive human serum and C) stained using a GADA positive serum from a patient with stiff person syndrome and D) canine serum from a dog with diabetes. Nuclear stain in blue (DAPI). Green is secondary anti rat Ig-FITC and anti-canine Ig-FITC used in A) and D). Red is anti-human Ig -Cy5 used in B) and D).

Using mouse monoclonal antibodies directed to rat GAD65, followed by fluorochrome-conjugated secondary antibodies directed to mouse IgG, the islets of Langerhans were visualized in pancreas from a healthy dog (Fig. 2a). Using sera from a GADA-positive human T1D patient (Fig. 2b) or a patient with stiff person syndrome (Fig. 2c) we were also able to stain the islets of Langerhans in the dog and human pancreata. Sera from dogs with diabetes mellitus and control dogs were in all cases unable to bind specifically (Fig. 2d). The level of unspecific binding was similar in samples from diabetic dogs and healthy control dogs. In addition, the certified human ICA assay was negative in all samples.

### GAD65 immunoprecipitations

**Figure 3:**
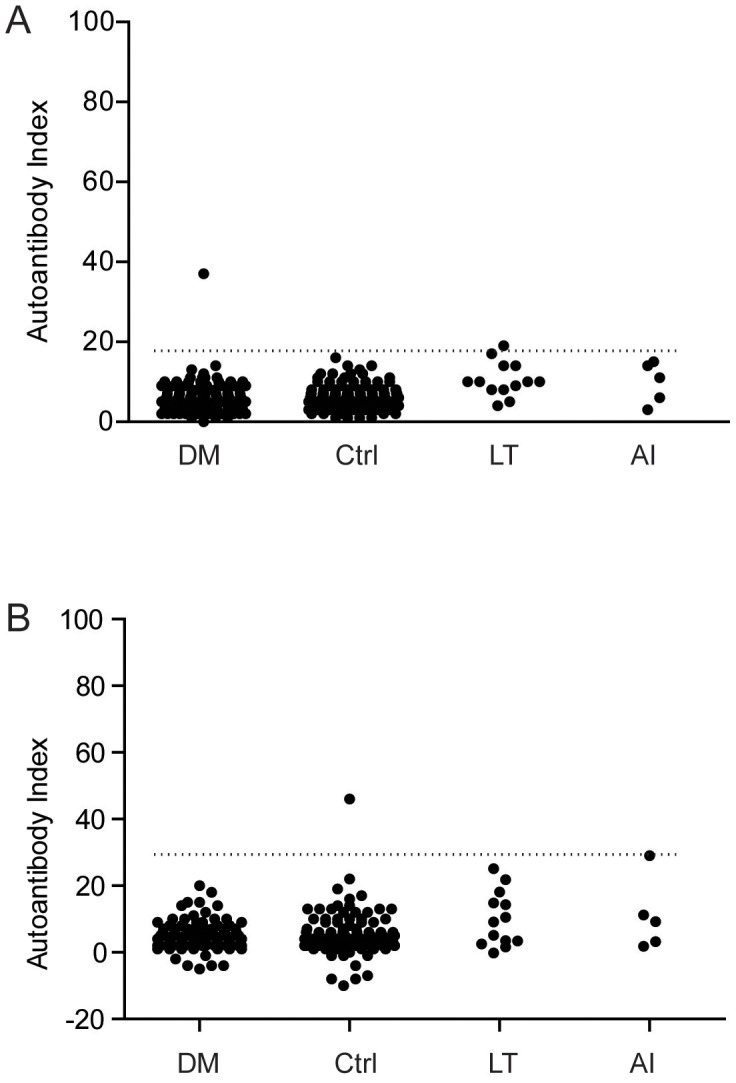
Immunoreactivity to A) Canine GAD65 and B) Human GAD65. DM =  diabetes mellitus, *n* = 121, Ctrl  =  healthy control, *n* = 133, LT  =  lymphocytic thyroiditis, *n* = 13 and AI  = adrenal insufficiency *n* = 5, Cut off index for positive vs. negative to GADA indicated by dotted line.

A strong titer of GAD65 autoantibodies was found in the human T1D serum used as a control, regardless whether canine or human GAD65 ITT products were used for immunoprecipitations. One diabetic Giant Schnauzer (0.8%) displayed weak reactivity against canine GAD65 (Fig. 2a) and one control Norwegian Elkhound (0.8%) was positive against human GAD65 (Fig. 2b). None of the dogs with lymphocytic thyroiditis or adrenal insufficiency were GADA-positive (Fig. 2a and 2b). The results are summarized in Fig. 3. Laboratory tests of the GADA-positive diabetic dog indicated a thyroid hormone deficiency, but the TSH level was normal (17 mIU/l). No further investigations of this dog were done as it was euthanized on request by the owner.

## Discussion

It has previously been suggested that the majority of canine diabetes mellitus is due to autoimmune T1D or latent autoimmune diabetes of the adult. We first used the ICA assay, which visualizes islet reactivity regardless of molecular target of autoantibodies to assess this hypothesis. The results from nearly all assays were negative, which is in line with a previous study, which reported that in 18 examined dogs with spontaneous diabetes mellitus, no islet-directed humoral autoimmunity was present [9]. Our findings were also supported by histopathologic examinations on pancreata obtained at the diagnosis of the disease and before start of insulin treatment, where no immune cells were seen in conjunction to the islets. The vacuolisation of some beta cells in combination with weak or absent insulin immune-staining may suggest that stress in the endoplasmic reticulum, so called ER-stress, due to a high demand for insulin and the consecutive protein misfolding [15]. There was a variety in the frequency of normal-appearing insulin-staining cells, even though time from onset of symptoms to euthanasia was similar among dogs. We speculate that the degree of insulin resistance in a dog will determine at what loss of beta cell mass the dog will go into a diabetic stage. Dogs are mainly carnivorous, and have fewer and smaller islets than humans. Even though some adaptation of the carbohydrate degradation pathways has occurred since divergence from the wolf [16], it may be speculated that a carbohydrate-rich diet may induce an ER-stress response if demand for insulin supersedes production capacity and that protein misfolding is part of the pathological mechanism in the development of canine diabetes mellitus.

A variety of diabetes autoantigens has been described in humans, however GAD65 is the most prevalent and specific autoantigen in human T1D. Therefore a GAD65 ITT analysis followed by immunoprecipitation was employed to investigate whether also diabetic dogs produce such autoantibodies. Because of the lack of canine positive control serum a human sample was tested and proved useful. As a precaution we tested both a novel canine GADA assay as well as an established human GADA assay. Our results are in opposition to a previous study where 4 of 30 dogs were GADA-positive and 3 of 30 dogs IA–2A positive [11]. The GADA-positive dogs were mongrels and springer spaniels, a breed also included in the present study. Furthermore, later the same group also reported the presence of antibodies to proinsulin in diabetic and control dogs using a Western blot technique. The implications of proinsulin autoantibodies are, however, unclear since three out of 15 (20%) of the control dogs included in that study were positive compared to 8/15 (53%) of newly diagnosed and 6/15 insulin-treated dogs (40%) [10]. The Swedish dog population is somewhat different from those of other Western countries, where female dogs are spayed early in life. The high proportion of intact female dogs in Sweden is associated with a female predisposition to diabetes mellitus [1], shown to be caused by progesterone-related diabetes mellitus [5]. Hence, the study material is not fully comparable to that of the UK study [11], which could explain some of the differences in results, although breeds were overlapping.

Rodent models such as the NOD mouse have shown that the primary autoantigen may differ between species [17].In the present study, we used the ICA to screen for other autoantibodies against canine pancreata than GAD-65, but all findings were negative. We cannot rule out cell-mediated autoimmunity, even though the histological appearance is in favor of other etiologies. Future studies should preferably investigate possible T-cell responses to islet cell autoantigens.

Assessing genetic variation in the major histocompatibility complex might have been valuable to test similarities to human risk HLA-DQ2 as done for dogs in [18], but such candidate gene approaches in dogs are at high risk of false positive findings unless population stratification bias is ruled out. Future studies should preferably investigate possible T-cell responses to islet cell autoantigens. Another important aspect of diabetes aetiology research in dogs is that dogs seem sensitive to glucose toxicity, and hyperglycemic dogs not treated immediately after diagnosis are at severe risk of permanent diabetes irrespective of the cause of hyperglycaemia [5], [19].

In conclusion, in this Swedish cohort of 121 diabetic dogs representing 40 different breeds, we find no ICA-positive canine sera, no insulitis and no differences between cases and controls in the GADA assay.

## Supporting Information

Figure S1 **Pancreatic specimens stained with hematoxylin-eosin (left column) and immunostained for insulin (middle column) or glucagon (right).** Images A-O show diabetic dogs and P-U healthy control dogs.
(TIF)

## References

[1] Fall T, Hamlin HH, Hedhammar A, Kampe O, Egenvall A (2007) Diabetes mellitus in a population of 180,000 insured dogs: incidence, survival, and breed distribution. J Vet Intern Med 21: 1209–1216. 18196728 10.1892/07-021.1

[2] Lindblad-Toh K, Wade CM, Mikkelsen TS, Karlsson EK, Jaffe DB, et al (2005) Genome sequence, comparative analysis and haplotype structure of the domestic dog. Nature 438: 803–819. 16341006 10.1038/nature04338

[3] Catchpole B, Ristic JM, Fleeman LM, Davison LJ (2005) Canine diabetes mellitus: can old dogs teach us new tricks? Diabetologia 48: 1948–1956. 16151773 10.1007/s00125-005-1921-1

[4] Rand JS, Fleeman LM, Farrow HA, Appleton DJ, Lederer R (2004) Canine and feline diabetes mellitus: nature or nurture? J Nutr 134: 2072S–2080S. 15284406 10.1093/jn/134.8.2072s

[5] Fall T, Hedhammar A, Wallberg A, Fall N, Ahlgren KM, et al (2010) Diabetes mellitus in elkhounds is associated with diestrus and pregnancy. J Vet Intern Med 24: 1322–1328. 21054539 10.1111/j.1939-1676.2010.0630.x

[6] Ling GV, Lowenstine LJ, Pulley LT, Kaneko JJ (1977) Diabetes mellitus in dogs: a review of initial evaluation, immediate and long-term management, and outcome. J Am Vet Med Assoc 170: 521–530. 557467

[7] Gepts W, Toussaint D (1967) Spontaneous diabetes in dogs and cats. A pathological study. Diabetologia 3: 249–265. 4190607 10.1007/BF01222202

[8] Eigenmann JE, Eigenmann RY, Rijnberk A, van der Gaag I, Zapf J, et al (1983) Progesterone-controlled growth hormone overproduction and naturally occurring canine diabetes and acromegaly. Acta Endocrinol (Copenh) 104: 167–176. 6227190 10.1530/acta.0.1040167

[9] Alejandro R, Feldman EC, Shienvold FL, Mintz DH (1988) Advances in canine diabetes mellitus research: etiopathology and results of islet transplantation. J Am Vet Med Assoc 193: 1050–1055. 3143693

[10] Davison LJ, Herrtage ME, Catchpole B (2011) Autoantibodies to recombinant canine proinsulin in canine diabetic patients. Res Vet Sci 91: 58–63. 20855094 10.1016/j.rvsc.2010.08.007

[11] Davison LJ, Weenink SM, Christie MR, Herrtage ME, Catchpole B (2008) Autoantibodies to GAD65 and IA-2 in canine diabetes mellitus. Vet Immunol Immunopathol 126: 83–90. 18706702 10.1016/j.vetimm.2008.06.016

[12] Hoenig M, Dawe DL (1992) A qualitative assay for beta cell antibodies. Preliminary results in dogs with diabetes mellitus. Vet Immunol Immunopathol 32: 195–203. 1632063 10.1016/0165-2427(92)90046-s

[13] Foulis AK, Liddle CN, Farquharson MA, Richmond JA, Weir RS (1986) The histopathology of the pancreas in type 1 (insulin-dependent) diabetes mellitus: a 25-year review of deaths in patients under 20 years of age in the United Kingdom. Diabetologia 29: 267–274. 3522324 10.1007/BF00452061

[14] Fall T, Holm B, Karlsson A, Ahlgren KM, Kampe O, et al (2008) Glucagon stimulation test for estimating endogenous insulin secretion in dogs. Vet Rec 163: 266–270. 18757903 10.1136/vr.163.9.266

[15] Gardner BM, Pincus D, Gotthardt K, Gallagher CM, Walter P (2013) Endoplasmic reticulum stress sensing in the unfolded protein response. Cold Spring Harb Perspect Biol 5: a013169. 23388626 10.1101/cshperspect.a013169 PMC3578356

[16] Axelsson E, Ratnakumar A, Arendt ML, Maqbool K, Webster MT, et al (2013) The genomic signature of dog domestication reveals adaptation to a starch-rich diet. Nature 495: 360–364. 23354050 10.1038/nature11837

[17] Nakayama M, Abiru N, Moriyama H, Babaya N, Liu E, et al (2005) Prime role for an insulin epitope in the development of type 1 diabetes in NOD mice. Nature 435: 220–223. 15889095 10.1038/nature03523 PMC1364531

[18] Kennedy LJ, Davison LJ, Barnes A, Short AD, Fretwell N, et al (2006) Identification of susceptibility and protective major histocompatibility complex haplotypes in canine diabetes mellitus. Tissue Antigens 68: 467–476. 17176436 10.1111/j.1399-0039.2006.00716.x

[19] Imamura T, Koffler M, Helderman JH, Prince D, Thirlby R, et al (1988) Severe diabetes induced in subtotally depancreatized dogs by sustained hyperglycemia. Diabetes 37: 600–609. 3282947 10.2337/diab.37.5.600

