# Supplementary figures and images for: Lack of Evidence for a Role of Islet Autoimmunity in the Aetiology of Canine Diabetes Mellitus
Source: PLoS One. 2014 Aug 25;9(8):e105473. doi: 10.1371/journal.pone.0105473 (PMC4143278; doi:10.1371/journal.pone.0105473)

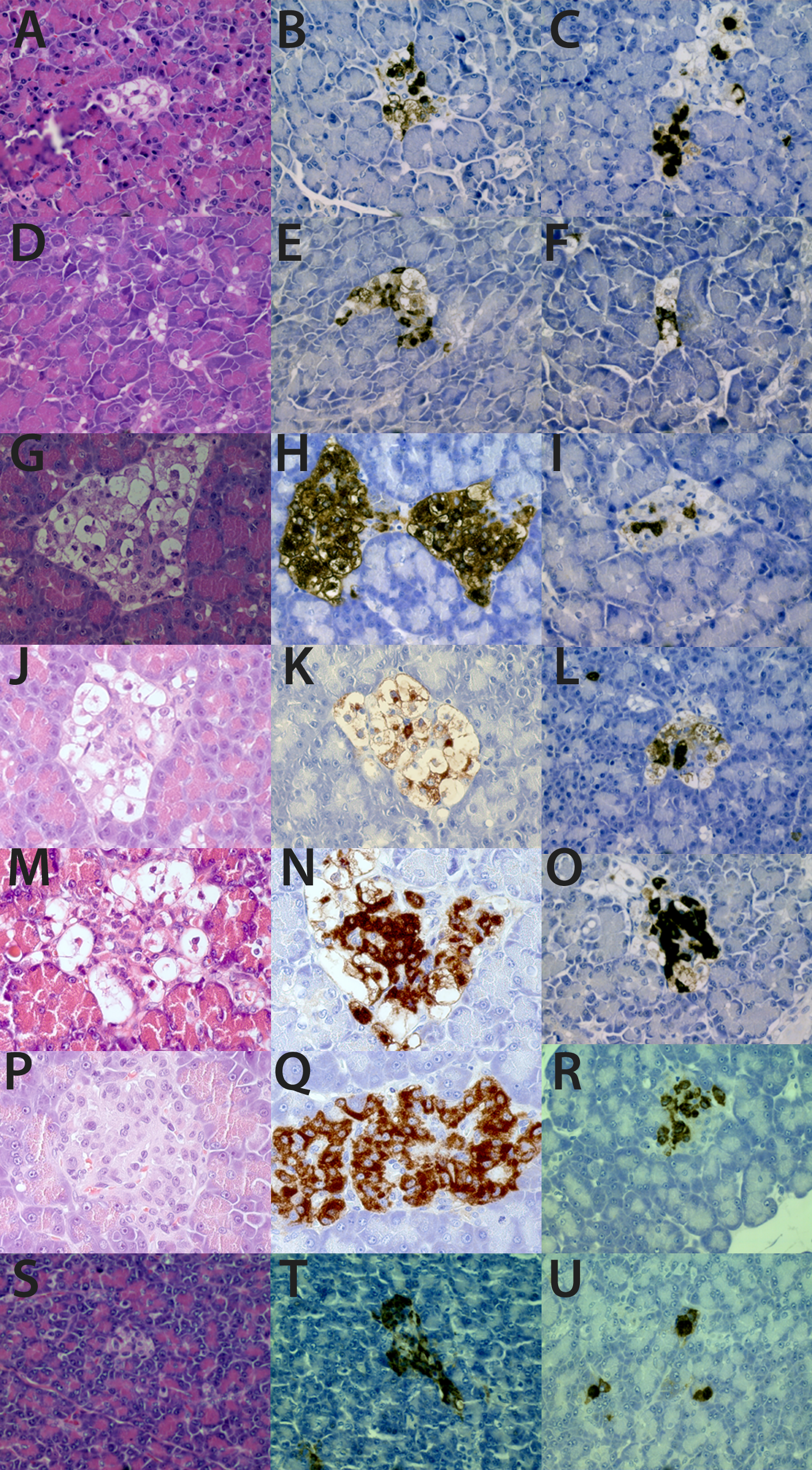

Supplement: Figure S1 — Pancreatic specimens stained with hematoxylin-eosin (left column) and immunostained for insulin (middle column) or glucagon (right). Images A-O show diabetic dogs and P-U healthy control dogs. (TIF) [file pone.0105473.s001.tif]
